# Incidence of Aggressive End-of-Life Care Among Older Adults With Metastatic Cancer Living in Nursing Homes and Community Settings

**DOI:** 10.1001/jamanetworkopen.2023.0394

**Published:** 2023-02-22

**Authors:** Siran M. Koroukian, Sara L. Douglas, Long Vu, Hannah L. Fein, Richa Gairola, David F. Warner, Nicholas K. Schiltz, Jennifer Cullen, Cynthia Owusu, Martha Sajatovic, Johnie Rose

**Affiliations:** 1 Department of Population and Quantitative Health Sciences, Case Western Reserve University School of Medicine, Cleveland, Ohio; 2 Case Comprehensive Cancer Center, Case Western Reserve University School of Medicine, Cleveland, Ohio; 3 Frances Payne Bolton School of Nursing, Case Western Reserve University, Cleveland, Ohio; 4 now with Department of Epidemiology, School of Public Health, Brown University, Providence, Rhode Island; 5 Department of Sociology, University of Alabama at Birmingham, Birmingham; 6 Center for Family and Demographic Research, Bowling Green State University, Bowling Green, Ohio; 7 Department of Internal Medicine, University Hospitals Cleveland Medical Center, Cleveland, Ohio; 8 Department of Neurology, University Hospitals Cleveland Medical Center, Cleveland, Ohio; 9 Center for Community Health Integration, Case Western Reserve University School of Medicine, Cleveland, Ohio

## Abstract

**Importance:**

Nearly 10% of the 1.5 million persons residing in nursing homes (NHs) have received or will receive a diagnosis of cancer. Although aggressive end-of-life (EOL) care is common among community-dwelling patients with cancer, little is known about such patterns of care among NH residents with cancer.

**Objective:**

To compare markers of aggressive EOL care between older adults with metastatic cancer who are NH residents and their community-dwelling counterparts.

**Design, Setting, and Participants:**

This cohort study used the Surveillance, Epidemiology, and End Results database linked with the Medicare database and the Minimum Data Set (including NH clinical assessment data) for deaths occurring from January 1, 2013, to December 31, 2017, among 146 329 older patients with metastatic breast, colorectal, lung, pancreas, or prostate cancer, with a lookback period in claims data through July 1, 2012. Statistical analysis was conducted between March 2021 and September 2022.

**Exposures:**

Nursing home status.

**Main Outcomes and Measures:**

Markers of aggressive EOL care were cancer-directed treatment, intensive care unit admission, more than 1 emergency department visit or more than 1 hospitalization in the last 30 days of life, hospice enrollment in the last 3 days of life, and in-hospital death.

**Results:**

The study population included 146 329 patients 66 years of age or older (mean [SD] age, 78.2 [7.3] years; 51.9% men). Aggressive EOL care was more common among NH residents than community-dwelling residents (63.6% vs 58.3%). Nursing home status was associated with 4% higher odds of receiving aggressive EOL care (adjusted odds ratio [aOR], 1.04 [95% CI, 1.02-1.07]), 6% higher odds of more than 1 hospital admission in the last 30 days of life (aOR, 1.06 [95% CI, 1.02-1.10]), and 61% higher odds of dying in the hospital (aOR, 1.61 [95% CI, 1.57-1.65]). Conversely, NH status was associated with lower odds of receiving cancer-directed treatment (aOR, 0.57 [95% CI, 0.55-0.58]), intensive care unit admission (aOR, 0.82 [95% CI, 0.79-0.84]), or enrollment in hospice in the last 3 days of life (aOR, 0.89 [95% CI, 0.86-0.92]).

**Conclusions and Relevance:**

Despite increased emphasis to reduce aggressive EOL care in the past several decades, such care remains common among older persons with metastatic cancer and is slightly more prevalent among NH residents than their community-dwelling counterparts. Multilevel interventions to decrease aggressive EOL care should target the main factors associated with its prevalence, including hospital admissions in the last 30 days of life and in-hospital death.

## Key Points

**Question:**

Is receipt of aggressive end-of-life (EOL) care for older adults with metastatic cancer more common among nursing home (NH) residents or their community-dwelling counterparts?

**Findings:**

This cohort study of 146 329 older adults found that aggressive EOL care was more common for NH residents than for community-dwelling residents (64% vs 58%). The key markers associated with the higher prevalence of aggressive EOL care were more than 1 hospital admission in the last 30 days of life and in-hospital death.

**Meaning:**

This study suggests that aggressive EOL care is widespread among older adults with metastatic cancer and more prevalent among NH residents than among community-dwelling patients.

## Teaser

This cohort study uses data from the Surveillance, Epidemiology, and End Results database to compare markers of aggressive end-of-life care between older adults with metastatic cancer who are nursing home residents and their community-dwelling counterparts.

## Introduction

In the United States, nursing home (NH) stays at the end of life (EOL) are common among older adults; 43.5% of Medicare fee-for-service beneficiaries have an NH stay in the last 90 days of life, and 25% die in an NH.^1^ In addition, nearly 150 000 of the more than 1.5 million persons residing in US NHs have received or will receive a diagnosis of cancer.^2^ The treatment of cancer and EOL care among NH residents are complicated by the fact that most institutionalized older adults present with a high morbidity burden, functional dependency, and/or cognitive impairment; many also experience adverse outcomes after cancer treatment.^3,4^ Although aggressive EOL care remains common among community-dwelling patients with cancer, little is known about such patterns of care among NH residents with cancer.^5^

The use of aggressive disease–oriented interventions at EOL has been associated with increasing symptom burden; frequent use of emergency department (ED), hospital, and intensive care unit (ICU) care; delay in accessing hospice programs; and enormous personal and financial costs. These patterns of aggressive treatment are considered key markers indicating poor quality of EOL care^6,7,8,9,10^ and are important factors associated with health care costs, which vary by region,^11^ patient characteristics,^11,12,13^ and patterns of care^14^; however, they offer little or no benefits relative to cure or survival. Instead, early enrollment in palliative care by patients with metastatic cancer—by emphasizing symptom management, illness understanding, and prognostic awareness^15^—is associated with less aggressive care, improvements in quality of life, longer survival,^16,17^ and reduced costs in the last month of life, primarily through reduced hospitalizations.^18^

Previous population-based studies on EOL care have not differentiated between community-dwelling older adults with cancer and their counterparts in NHs. This is an important gap in the literature because it limits our understanding of EOL care among a very vulnerable group of patients with cancer—those residing in NHs with varied abilities to participate in their own EOL decision-making due to cognitive impairment.

We address this gap in the literature using a unique data resource, the Surveillance, Epidemiology, and End Results (SEER) database linked with Medicare enrollment and claims data and the Minimum Data Set (MDS),^19,20^ which includes NH clinical assessment data. We compare markers of aggressive EOL care for older adults with metastatic cancer who died in 2013 to 2017 between those who were community dwelling and their NH counterparts. We hypothesize that, compared with community-dwelling older adults with cancer, aggressive EOL care is less common among NH residents, given their complex health care needs and the questionable benefits associated with aggressive interventions.

## Methods

### Data Sources

We used the linked SEER-Medicare-MDS database for July 1, 2012, to December 31, 2017, for deaths occurred during calendar years 2013 to 2017. The 6-month look-back period in 2012 allowed us to flag comorbid conditions, treatment received, and other health services utilization measures before death. The SEER program of the National Cancer Institute contains authoritative data from population-based cancer registries for individuals with incident cancer cases who reside within each registry’s select geographic catchment areas, covering nearly 48% of the US population.^21^ In addition to sociodemographic data (eg, age, sex, and race and ethnicity) and month and year of cancer diagnosis, the SEER database includes tumor characteristics, treatment modalities (eg, surgery and radiotherapy), and date and cause of death for those who have died. This study was approved by the Case Western Reserve University institutional review board, which did not require patient consent. This study followed the Strengthening the Reporting of Observational Studies in Epidemiology (STROBE) reporting guideline for observational studies.

The linked database contains Medicare enrollment and claims data for patients in the SEER database. The Medicare Beneficiary Summary File (MBSF) represents all Medicare beneficiaries, regardless of whether they receive their care through managed care programs or through the traditional fee-for-service system. In addition to demographic data, the MBSF includes monthly indicators for individuals’ enrollment in Medicare Parts A, B, C, and D and the dual Medicare and Medicaid program. The claims data are included in the Medicare Provider, Analysis, and Review (MedPAR) for inpatient hospital admissions and standard analytic files for outpatient institutional and noninstitutional care, hospice care, durable medical equipment, and prescription drugs. Claims data were used to identify aggressive EOL care indicators.

The MDS (version 3.0) is part of a federally mandated process for clinical assessment of all residents in Medicare- or Medicaid-certified NHs.^22^ The MDS consists of clinical assessment data for all NH residents that are completed on admission, at discharge, and on a quarterly basis (or, more often, if deemed clinically necessary by NH staff). Minimum Data Set data include a detailed assessment of individuals’ cognitive and functional status, mood and behavioral symptoms, medications, therapies (including hospice, chemotherapy, and radiotherapy), and treatments received through NH care. The linkage of these data sets is accomplished collaboratively by the National Cancer Institute, SEER registries, and the Centers for Medicare & Medicaid Services.

### Study Population

The study population included individuals aged 66 years or older at their time of cancer diagnosis; identified in claims data for services received during the last 6 months of life for active metastatic female breast, colorectal, lung, pancreas, or prostate cancer; and who died (from any cause of death) between 2013 and 2017. We restricted our study population to these 5 cancer types because, combined, they account for nearly half of all incident cancer cases in the US.^23^ Patients with metastatic cancer were identified based on the presence of relevant diagnosis codes, as specified by the Elixhauser Comorbidity Index.^24^

To ensure complete Medicare claims history at EOL, we limited our final study cohort to patients receiving their care through the traditional fee-for-service Medicare system in the 6 months preceding death, which required our use of Medicare data dating back to July 1, 2012, as noted earlier. Last, we excluded individuals with unknown or missing race and ethnicity data from our study cohort, yielding a final analytic cohort of 146 329 individuals (eFigure 1 in Supplement 1).

### Variables of Interest

#### Outcome Variables

The primary outcome of interest in this study was receipt of aggressive EOL care, defined by the presence of any of the following markers in the last 30 days of life: any cancer-directed treatment modality (including surgery, radiotherapy, or chemotherapy), more than 1 ED visit, more than 1 hospital admission, and any ICU admission. Additional markers were hospice entry in the last 3 days of life based on the first date of service in the first hospice claim relative to the date of death, and death in the hospital.^7,8,9,25^ All variables for markers for aggressive EOL care were derived from Medicare claims data, including indicators of chemotherapy and cancer-directed surgery and radiotherapy (see eTables 1 and 2 in Supplement 1 for all codes used to define markers).

#### Independent Variables

Our main independent variable of interest was NH status, defined by the presence of at least 1 comprehensive MDS assessment in the 6 months prior to death. Other key covariates included age at death (categorized into groups of 66-74 years, 75-84 years, or ≥85 years); sex (male or female); and race and ethnicity (Hispanic [all races], non-Hispanic American Indian or Alaska Native, Non-Hispanic Asian or Pacific Islander, non-Hispanic Black, or non-Hispanic White), collected by the SEER from various sources, including patient report and medical records,^26,27^ and recoded using SEER-recommended categories combining Hispanic ethnicity with race data.^10^ We accounted for race and ethnicity to analyze patterns of EOL care across the different racial and ethnic groups. Additional covariates were Medicare and Medicaid dual eligibility status in the last 6 months of life (proxy for socioeconomic status); Elixhauser comorbidity^24^ count in the final 6 months of life (categorized as ≤4 or ≥5 Elixhauser comorbidities), based on the observed distribution of NH and community-dwelling patients by number of comorbid conditions in our data^11^; and cancer type (lung, breast, colorectal, pancreatic, or prostate). Exact date of death was extracted from MBSF enrollment files or the MedPAR records. If unavailable from these sources, date of death was imputed to the middle of the month using month and year data from the SEER database.

These variables were added as covariates in our multivariable models. Based on prior studies, we hypothesized that being younger, a person from a racial or ethnic minority group,^28,29,30^ or dually enrolled in Medicare and Medicaid^31^ would be associated with higher odds of receiving aggressive EOL care and that having a higher comorbidity burden or a cancer with high case-fatality rate (eg, lung or pancreas cancer) would be associated with lower odds of receiving aggressive EOL care. Last, we added the year of death variable to detect potential temporal trends in the implementation of Patient Protection and Affordable Care Act (ACA)–related stipulations to improve EOL care,^32^ even if the study years were in the post-ACA era.

### Statistical Analysis

Statistical analysis was conducted between March 2021 and September 2022. In addition to descriptive analyses, we conducted bivariate analyses to assess differences in receipt of markers for aggressive EOL care by NH status. We subsequently developed multivariable logistic regression models to evaluate the association between NH status and aggressive EOL care after adjusting for potential confounders already listed. We used 2-sided tests for statistical significance. For sensitivity analysis, we restricted NH residents to only those with confirmed enrollment in an NH within their last 30 days of life.

In comparisons, we refrained from presenting *P* values to indicate statistical significance due to the large sample size,^33,34^ focusing instead on the clinical meaningfulness of the observed differences between the 2 comparison groups: NH residents and their community-dwelling counterparts. All statistical programming and analyses were completed using SAS, version 9.4 (SAS Institute, Inc) and R, version 4.1.1 (R Group for Statistical Computing).

## Results

As shown in Table 1, our study population included 146 329 older adults (mean [SD] age, 78.2 [7.3] years; 51.9% men) with active metastatic breast, colorectal, lung, pancreas, or prostate cancer; of those, 28.0% were NH residents. Community-dwelling patients were younger than NH residents (median age, 77 years [IQR, 72-83 years] vs 79 years [IQR, 74-85 years]). The distribution of patients with cancer by sex and race and ethnicity was generally comparable between the 2 groups, although there was a slightly higher percentage of women and non-Hispanic Black individuals in NHs relative to the community (women, 50.8% vs 47.1%; non-Hispanic Black individuals, 10.5% vs 8.1%). Compared with community-dwelling patients, those in an NH included a higher percentage of individuals dually eligible for Medicare and Medicaid (30.4% vs 17.1%) and individuals with at least 5 comorbid conditions (75.5% vs 48.8%). Conversely, the percentage of patients with high case-fatality cancers was higher among the community-dwelling group than among NH residents (lung cancer, 46.0% vs 43.0%; pancreatic cancer, 14.3% vs 9.3%).

**Table 1.** Patient Demographic Characteristics Stratified by NH Status Among Medicare FFS Enrollees Aged 66 Years or Older Who Died Between 2013 and 2017 With Active Metastatic Cancer in Their Last 6 Months of Life

Characteristic	Patients, No. (%)		
	Total (N = 146 329)	NH (n = 40 965)	Community dwelling (n = 105 364)
Age group, y			
66-74	52 399 (35.8)	11 747 (28.7)	40 652 (38.6)
75-84	62 586 (42.8)	17 964 (43.9)	44 622 (42.3)
≥85	31 344 (21.4)	11 254 (27.5)	20 090 (19.1)
Mean (SD)	78.2 (7.3)	79.6 (7.4)	77.6 (7.2)
Median (IQR)	77 (72-84)	79 (74-85)	77 (72-83)
Sex			
Male	75 909 (51.9)	20 166 (49.2)	55 743 (52.9)
Female	70 420 (48.1)	20 799 (50.8)	49 621 (47.1)
Race and ethnicity			
Hispanic	8157 (5.6)	2055 (5.0)	6102 (5.8)
Non-Hispanic			
American Indian or Alaska Native	540 (0.4)	132 (0.3)	408 (0.4)
Asian or Pacific Islander	6671 (4.6)	1767 (4.3)	4904 (4.7)
Black	12 865 (8.8)	4300 (10.5)	8565 (8.1)
White	118 096 (80.7)	32 711 (79.9)	85 385 (81.0)
Dual eligible status^a^			
No	115 847 (79.2)	28 502 (69.6)	87 345 (82.9)
Yes	30 482 (20.8)	12 463 (30.4)	18 019 (17.1)
Cancer type			
Lung	66 106 (45.2)	17 562 (43.0)	48 544 (46.0)
Breast	15 550 (10.6)	5139 (12.5)	10 411 (9.9)
Colorectal	23 145 (15.8)	7182 (17.5)	15 963 (15.2)
Pancreatic	18 904 (12.9)	3816 (9.3)	15 088 (14.3)
Prostate	22 624 (15.5)	7266 (17.7)	15 358 (14.6)
Year of death			
2013	29 085 (19.9)	7657 (18.7)	21 428 (20.3)
2014	29 133 (19.9)	8292 (20.2)	20 841 (19.8)
2015	28 947 (19.8)	8319 (20.3)	20 628 (19.6)
2016	29 601 (20.2)	8461 (20.7)	21 140 (20.1)
2017	29 563 (20.2)	8236 (20.1)	21 327 (20.2)
Elixhauser comorbidity count			
≤4	64 021 (43.8)	10 050 (24.5)	53 971 (51.2)
≥5	82 308 (56.3)	30 915 (75.5)	51 393 (48.8)
Mean (SD)	5.1 (2.7)	6.4 (2.7)	4.6 (2.5)
Median (IQR)	5.0 (3-7)	6.0 (5-8)	4.0 (3-6)

Abbreviations: FFS, fee-for-service; NH, nursing home.

^a^ Eligible for both Medicare and Medicaid.

A total of 58.3% of patients in the community-dwelling group and 63.6% of NH residents received aggressive EOL care (Table 2). This percentage varied by demographic characteristics, dual eligibility status, cancer type, and number of comorbid conditions (Figure). Along with variations in aggressive EOL care by these patient characteristics, we observed a higher percentage of aggressive EOL care among NH residents than among community-dwelling residents.

**Table 2.** Analysis of Markers for Receipt of Aggressive EOL Care by Nursing Home Status

EOL indicator	Patients, No. (%)			Odds ratio (95% CI)	
	Total (N = 146 329)	Nursing home (n = 40 965)	Community dwelling (n = 105 364)	Unadjusted	Adjusted
Any aggressive EOL care	87 454 (59.8)	26 046 (63.6)	61 408 (58.3)	1.25 (1.22-1.28)	1.04 (1.02-1.07)
Any cancer-directed treatment	38 160 (26.1)	7678 (18.7)	30 482 (28.9)	0.57 (0.55-0.58)	0.57 (0.55-0.58)
>1 ED visit	30 738 (21.0)	9374 (22.9)	21 364 (20.3)	1.17 (1.14-1.20)	0.97 (0.95-1.00)
>1 Hospital admission	17 952 (12.3)	5874 (14.3)	12 078 (11.5)	1.29 (1.25-1.34)	1.06 (1.02-1.10)
Any ICU admission	31 561 (21.6)	8760 (21.4)	22 801 (21.6)	0.98 (0.96-1.01)	0.82 (0.79-0.84)
Hospice entry in last 3 d of life	20 449 (14.0)	5494 (13.4)	14 955 (14.2)	0.94 (0.91-0.97)	0.89 (0.86-0.92)
Death in hospital	42 359 (29.0)	15 966 (39.0)	26 393 (25.1)	1.91 (1.87-1.96)	1.61 (1.57-1.65)

Abbreviations: ED, emergency department; EOL, end-of-life; ICU, intensive care unit.

**Figure.**
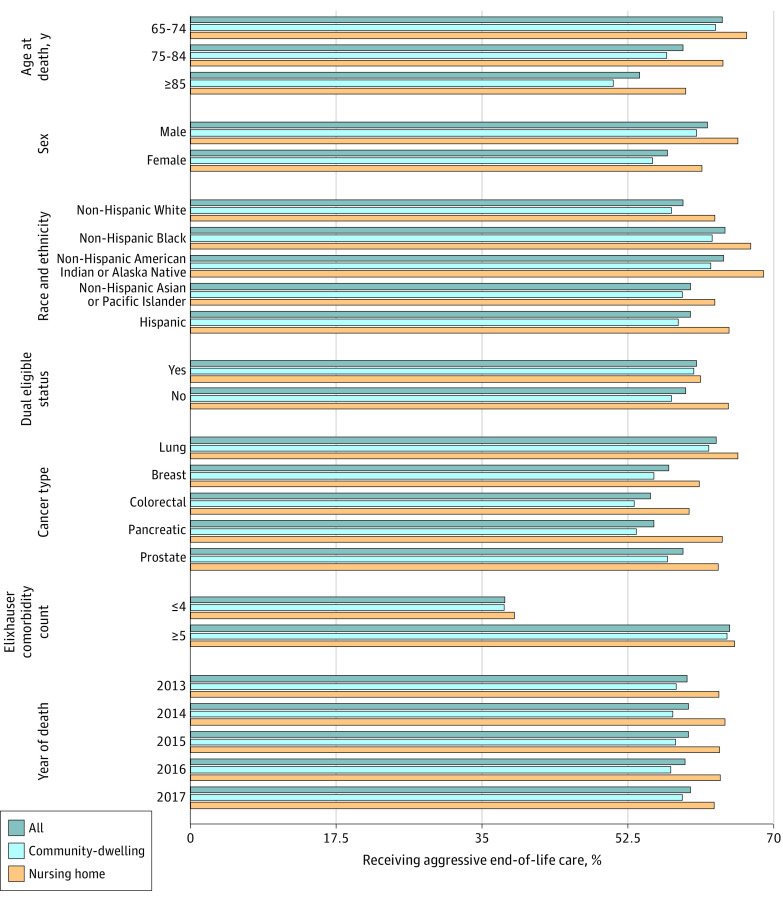
Comparison of Receipt of Aggressive End-of-Life Care by Age at Death, Sex, Race and Ethnicity, Dual Eligible Medicare and Medicaid Status, Elixhauser Comorbidity Count, Cancer Type, and Year of Death

As shown in Table 2, indicators of aggressive EOL care that were more common among NH residents than among community-dwelling older adults included more than 1 ED visit (22.9% vs 20.3%), more than 1 hospitalization (14.3% vs 11.5%), and in-hospital death (39.0% vs 25.1%). A lower percentage of NH residents than community-dwelling patients received cancer-directed treatment (18.7% vs 28.9%). The percentage of individuals entering hospice in the last 3 days of life was somewhat lower among NH residents than among their community-dwelling counterparts (13.4% vs 14.2%), and there was no notable difference in ICU admission between the 2 groups (NH residents, 21.4%; community-dwelling individuals, 21.6%).

Results from the multivariable regression analysis (Table 2) indicate that NH status was associated with 4% higher odds of receiving any marker of aggressive EOL care (adjusted odds ratio [aOR], 1.04 [95% CI, 1.02-1.07]), 6% higher odds of more than 1 hospital admission in the last 30 days of life (aOR, 1.06 [95% CI, 1.02-1.10]), and 61% higher odds of dying in the hospital (aOR, 1.61 [95% CI, 1.57-1.65]). Nursing home status was no longer significantly associated with more than 1 ED visit after adjusting for comorbid conditions (aOR, 0.97 [95% CI, 0.95-1.00]). Nursing home status was associated with nearly half the odds of receiving cancer-directed treatment in the last 30 days of life (aOR, 0.57 [95% CI, 0.55-0.58]), lower odds of being admitted to the ICU (aOR, 0.82 [95% CI, 0.79-0.84]), or lower odds of enrolling in hospice in the last 3 days of life (aOR, 0.89 [95% CI, 0.86-0.92]).

Compared with non-Hispanic White individuals, patients from other racial and ethnic groups had higher odds of receiving aggressive EOL care. Asian and Pacific Islander individuals had 33% higher odds (aOR, 1.33 [95% CI, 1.26-1.41]) and non-Hispanic Black individuals had 18% higher odds (aOR, 1.18 [95% CI, 1.13-1.23]) than non-Hispanic White individuals of dying in the hospital (eTable 3 in Supplement 1). In addition, while dually eligible patients had lower odds than non–dually eligible individuals of receiving aggressive EOL care (aOR, 0.92 [95% CI, 0.90-0.95]), they had higher odds of dying in a hospital (aOR, 1.10 [95% CI, 1.07-1.13]). The period effect varied depending on the aggressive EOL care marker. In particular, we noted decreased odds of receipt of cancer-directed treatment (aOR, 0.86 [95% CI, 0.83-0.90]) and in-hospital death (aOR, 0.95 [95% CI, 0.91-0.98]) in 2017 compared with 2013 but higher odds of having more than 1 ED visit (aOR, 1.15 [95% CI, 1.11-1.20]), ICU admission (aOR, 1.06 [95% CI, 1.02-1.11]), and late entry in hospice (aOR, 1.06 [95% CI, 1.01-1.11]). Last, patients with a higher comorbidity burden had higher odds of receiving aggressive EOL care for all markers, including nearly 2.5 times the odds of receiving aggressive EOL care (any marker: aOR, 2.42 [95% CI, 2.37-2.47]) compared with those with a lower comorbidity burden and nearly 3.5 times the odds of having more than 1 hospital admission in the last 30 days of life (aOR, 3.43 [95% CI, 3.30-3.57]).

### Sensitivity Analyses

We compared our results before and after defining NH status in the last month of life with the last 6 months of life. In every instance, the effect size for NH status was larger in the latter scenario (eFigure 2 in Supplement 1).

## Discussion

Using population-based data from the SEER database, Medicare, and the MDS for deaths that occurred from 2013 to 2017, we compared patterns of aggressive EOL care between community-dwelling older adults with cancer and their NH counterparts. Contrary to our hypothesis, our findings showed that aggressive EOL care among older adults with cancer was more common among NH residents than among their community-dwelling counterparts and was associated with hospital admissions in the last 30 days of life and in-hospital death.

Notwithstanding our findings specific to NHs, our results, including those showing differences in EOL care by race and ethnicity^28,29,30^ or by dual eligibility status,^30,35^ are consistent with previous studies. These patterns of aggressive EOL care, especially for NH residents, many of whom have a high comorbidity burden, stand in contrast with the increased emphasis over the past decades on reducing aggressive EOL care. The literature is replete with relevant interventions, such as the ones based on inpatient palliative care consultations and inpatient palliative care units, that have been shown to be effective in reducing hospitalization costs.^36,37,38^ The reasons for aggressive EOL care are multifactorial,^39^ including family involvement, religion and spirituality, patient preferences,^40^ patient-clinician communication,^41,42,43^ and health care delivery systems.^31,44^ For example, health systems with no cancer center or integrated affiliation are associated with higher proportions of patients with more than 1 hospitalization or lack of hospice use in the last year of life or death in an acute setting.^31,45^

### Strengths and Limitations

Our study has several strengths. To our knowledge, this is the first study to compare aggressive EOL care among older persons with cancer by NH status. With higher use of inpatient care associated with the difference in EOL care overall, our findings point to a heavy reliance of NHs on hospital care, even when patients have metastatic cancer and as patients are approaching death. Other strengths include our use of the linked SEER-Medicare-MDS database, allowing us to identify patients with metastatic cancer, derive markers of aggressive EOL care, and ascertain NH status among older patients with cancer. Last, our large study population allowed us to compare our outcomes of interest across various demographic and clinical strata.

Our findings should be interpreted considering the following limitations. First, it was not possible to describe the circumstances surrounding receipt of aggressive EOL care and whether care decisions were made in accordance with the patient’s preferences (or with those of their proxies if the patient was unable to participate in decision-making). Related to this limitation was the absence of data on advance care planning in this version of the MDS, which contributed to our inability to assess whether discussions relevant to EOL care with the patient and the proxies had taken place, and what the focus of directives were should the patient’s condition worsen. Nonetheless, the fact that receipt of cancer-directed treatment was lower among NH residents than among their community-dwelling counterparts reflects a decision—whether by the patient or proxies and/or health care professional—to decrease care. However, the reasons why such a decision was not coupled with early enrollment in hospice remain unaddressed. A better understanding of these circumstances requires a detailed qualitative study to gain insight into the decisions surrounding hospital transfer, from the perspectives of both the patient or their proxies and the NH. Second, contrary to decisions pertaining to receipt of cancer treatment, enrollment in hospice and palliative care may require coordinated decisions, including collaboration within and between organizations, as well as among hospital departments and family members. These activities are often perceived to be resource consuming,^46^ posing a “managerial challenge.”^47^ This perception is reflected in our data, showing higher odds of receiving most types of aggressive EOL care, except for cancer-directed treatment. Third, a lack of information on any advance care plan that may have been lost as NH patients were transferred to the hospital, the availability of family to assist in decisions regarding hospice or palliative care, and the accessibility of such services locally were all factors that may have been associated with use of palliative care, as prior research has shown that EOL conversations promote palliative care.^43^ Fourth, our data also did not include measures on functional dependence and social support, which are key potential confounders associated with both the need for NH care and the outcomes measured in this study. In addition, our study population was limited to patients who had claims documenting the presence of metastatic disease in the months preceding death. Fifth, this study was limited to US patients with cancer, and the results may not be generalizable to patients with cancer in other countries. Although NH services differ across countries worldwide, it is also recognized that how health care professionals from different services (eg, oncology, social work, and primary care) collaborate, share information, and construct cohesive plans of care likely differs significantly as well.^47,48^

## Conclusions

This study found that, despite the increased emphasis on reducing aggressive EOL care, such care remains highly prevalent among older persons with metastatic cancer and is more common among NH residents than their community-dwelling counterparts. Interventions to decrease aggressive EOL care should target the main factors associated with its prevalence, including hospital admissions in the last 6 months of life and in-hospital death.

## Notes

Supplement 1. **eTable 1.** Codes Used to Define End-of-Life Indicators **eTable 2.** Procedure Codes Used to Define Cancer-Directed Treatments **eTable 3.** Multivariable Logistic Regression Analysis for Receipt of Aggressive EOL With Adjusted Odds Ratios (OR) and 95% Confidence Intervals **eFigure 1.** Cohort Selection Flowchart With Exclusion/Inclusion Criteria **eFigure 2.** Sensitivity Analysis of Markers for Receipt of Aggressive EOL Care After Restricting NH Cohort to Only Those With Confirmed NH Enrollment in Last Month of Life

Supplement 2.
Data Sharing Statement
