# Supplementary material for: Incidence of Aggressive End-of-Life Care Among Older Adults With Metastatic Cancer Living in Nursing Homes and Community Settings
Source: JAMA Netw Open. 2023 Feb 22;6(2):e230394. doi: 10.1001/jamanetworkopen.2023.0394 (PMC9947721; doi:10.1001/jamanetworkopen.2023.0394)
Supplement: Supplement 1. — eTable 1. Codes Used to Define End-of-Life Indicators eTable 2. Procedure Codes Used to Define Cancer-Directed Treatments eTable 3. Multivariable Logistic Regression Analysis for Receipt of Aggressive EOL With Adjusted Odds Ratios (OR) and 95% Confidence Intervals eFigure 1. Cohort Selection Flowchart With Exclusion/Inclusion Criteria eFigure 2. Sensitivity Analysis of Markers for Receipt of Aggressive EOL Care After Restricting NH Cohort to Only Those With Confirmed NH Enrollment in Last Month of Life [file jamanetwopen-e230394-s001.pdf]

Supplementary Online Content

Koroukian SM, Douglas SL, Vu L, et al. Incidence of aggressive end-of-life care among older adults with metastatic cancer living in nursing homes and community settings. *JAMA Netw Open.* 2023;6(2):e230394. doi:10.1001/jamanetworkopen.2023.0394

eTable 1. Codes Used to Define End-of-Life Indicators
eTable 2. Procedure Codes Used to Define Cancer-Directed Treatments
eTable 3. Multivariable Logistic Regression Analysis for Receipt of Aggressive EOL With Adjusted Odds Ratios (OR) and 95% Confidence Intervals
eFigure 1. Cohort Selection Flowchart With Exclusion/Inclusion Criteria
eFigure 2. Sensitivity Analysis of Markers for Receipt of Aggressive EOL Care After Restricting NH Cohort to Only Those With Confirmed NH Enrollment in Last Month of Life

This supplementary material has been provided by the authors to give readers additional information about their work.

© 2023 Koroukian SM et al. *JAMA Network Open.*

eTable 1. Codes Used to Define End-of-Life Indicators

**EOL Indicator**	**Codes**
Any Cancer-Directed Treatment	See supplemental table 2 for complete list.
>1 Emergency Department Visit	**Revenue center codes:**<br>"0450","0451","0452","0453","0454","0455","0456","0457","0459","0981"<br><br>**CPT codes:**<br>"99281","99282","99283","99284","99285"
>1 Hospital Admission	Count of MedPAR records with different billing dates in last 30 days of life, excluding SNF stays
Any ICU Admission	**Revenue center codes:**<br>"0200","0201","0202","0203","0204","0207","0208","0209"
Hospice Entry <3 Days of Life	Hospice start date variable in the Medicare Hospice file
Death in Hospital	Reported discharge date of final MedPAR record equals date of death, excluding SNF stays

© 2023 Koroukian SM et al. *JAMA Network Open.*

eTable 2. Procedure Codes Used to Define Cancer-Directed Treatments

Cancer	Procedure type	ICD-9-CM	ICD-10-PCS	CPT/HCPCS
Lung	Biopsy	33.24–33.29, 33.3x, 34.02–34.09, 34.1x, 34.20-34.27	0B933ZX, 0B934ZX, 0B937ZX , 0B938ZX, 0B943ZX , 0B944ZX, 0B947ZX , 0B948ZX, 0B953ZX , 0B954ZX, 0B957ZX , 0B958ZX , 0B963ZX, 0B964ZX , 0B967ZX, 0B968ZX , 0B973ZX, 0B974ZX, 0B977ZX, 0B978ZX, 0B983ZX, 0B984ZX , 0B987ZX , 0B988ZX, 0B993ZX , 0B994ZX, 0B997ZX, 0B998ZX, 0B9B3ZX, 0B9B4ZX, 0B9B7ZX, 0B9B8ZX, 0BB33ZX, 0BB34ZX, 0BB37ZX, 0BB38ZX, 0BB43ZX, 0BB44ZX, 0BB47ZX, 0BB48ZX , 0BB53ZX, 0BB54ZX, 0BB57ZX, 0BB58ZX, 0BB63ZX, 0BB64ZX, 0BB67ZX, 0BB68ZX, 0BB73ZX, 0BB74ZX, 0BB77ZX, 0BB78ZX, 0BB83ZX, 0BB84ZX, 0BB87ZX, 0BB88ZX, 0BB93ZX, 0BB94ZX, 0BB97ZX, 0BB98ZX, 0BBB3ZX, 0BBB4ZX, 0BBB7ZX, 0BBB8ZX, 0BD34ZX, 0BD38ZX, 0BD44ZX, 0BD48ZX, 0BD54ZX, 0BD58ZX, 0BD64ZX, 0BD68ZX, 0BD74ZX, 0BD78ZX, 0BD84ZX, 0BD88ZX, 0BD94ZX, 0BD98ZX, 0BDB4ZX, 0BDB8ZX , 0B930ZX, 0B940ZX, 0B950ZX, 0B960ZX , 0B970ZX, 0B980ZX, 0B990ZX, 0B9B0ZX, 0BB30ZX , 0BB40ZX, 0BB50ZX, 0BB60ZX, 0BB70ZX, 0BB80ZX, 0BB90ZX, 0BBB0ZX, 0B9C3ZX, 0B9C4ZX, 0B9C7ZX , 0B9D3ZX, 0B9D4ZX, 0B9D7ZX, 0B9F3ZX, 0B9F4ZX, 0B9F7ZX, 0B9G3ZX, 0B9G4ZX, 0B9G7ZX, 0B9H3ZX, 0B9H4ZX, 0B9H7ZX, 0B9J3ZX, 0B9J4ZX, 0B9J7ZX, 0B9K3ZX, 0B9K4ZX, 0B9K7ZX, 0B9L3ZX, 0B9L4ZX, 0B9L7ZX, 0B9M3ZX, 0B9M4ZX, 0B9M7ZX, 0BBC3ZX, 0BBD3ZX, 0BBF3ZX, 0BBG3ZX, 0BBH3ZX, 0BBJ3ZX, 0BBK3ZX, 0BBL3ZX, 0BBM3ZX, 0BDC8ZX, 0BDD8ZX, 0BDF8ZX, 0BDG8ZX, 0BDH8ZX, 0BDJ8ZX, 0BDK8ZX, 0BDL8ZX, 0BDM8ZX, 0B9K8ZX, 0B9L8ZX, 0B9M8ZX, 0BBK7ZX, 0BBK8ZX, 0BBL7ZX, 0BBL8ZX , 0BBM7ZX, 0BBM8ZX, 0B9K0ZX, 0B9L0ZX, 0B9M0ZX, 0BBK0ZX, 0BBL0ZX, 0BBM0ZX, 0BJ00ZZ, 0BJ10ZZ, 0BJK0ZZ, 0BJL0ZZ, 01520ZZ, 01523ZZ, 01524ZZ, 3E0L3SF, 3E0L7SF , 3E0M3SF, 0WU807Z, 0WU80JZ, 0WU80KZ, 0WU847Z, 0WU84JZ, 0WU84KZ, 02JA0ZZ, 0WJC0ZZ, 0W390ZZ, 0W3B0ZZ, 0W3D0ZZ, 0W3Q0ZZ, 0W9930Z, 0W9B30Z , 0W190JG, 0W194JG, 0W1B0JG, 0W1B4JG, 0W9940Z, 0W994ZZ, 0W9B40Z, 0W9B4ZZ, 0B9N0ZZ, 0B9N80Z, 0B9P0ZZ , 0B9P80Z, 0BHQ0YZ, 0BHQ3YZ, 0BHQ4YZ, 0BHQ7YZ, 0BHQ8YZ, 0BPQ0YZ, 0BPQ3YZ, 0BPQ4YZ, 0BPQ7YZ, 0BPQ8YZ, 0BWQ0YZ, 0BWQ3YZ, 0BWQ4YZ, 0BWQ7YZ, 0BWQ8YZ, 0W9900Z, 0W990ZZ, 0W9B00Z, 0W9B0ZZ, 0WC90ZZ, 0WC93ZZ, 0WC94ZZ, 0WCB0ZZ, 0WCB3ZZ, 0WCB4ZZ, 0W9C00Z, 0W9C0ZZ, 0W9C40Z, 0W9C4ZZ, 0WCC0ZZ ,	31625, 31628, 31629, 32095, 32405, 32602, 32606, 39000, 39010, 39400

© 2023 Koroukian SM et al. *JAMA Network Open.*

			0WCC3ZZ, 0WCC4ZZ, 0BBN4ZX, 0BBP4ZX, 0BJ04ZZ, 0WJQ4ZZ, 0WJC4ZZ, 0WJD4ZZ, 0W980ZX, 0W983ZX, 0W984ZX, 0WB80ZX, 0WB83ZX, 0WB84ZX, 0WB8XZX, 0B9N0ZX, 0B9N3ZX, 0B9N4ZX, 0B9N8ZX, 0B9P0ZX, 0B9P3ZX, 0B9P4ZX, 0B9P8ZX, 0BBN0ZX, 0BBN3ZX, 0BBN8ZX, 0BBP0ZX, 0BBP3ZX, 0BBP8ZX, 0W990ZX, 0W993ZX, 0W994ZX, 0W9B0ZX, 0W9B3ZX, 0W9B4ZX, 0W9C3ZX, 0W9C4ZX, 0WBC3ZX, 0WBC4ZX, 0W9C0ZX, 0WBC0ZX , 0B9T0ZX, 0B9T3ZX , 0B9T4ZX, 0BBT0ZX, 0BBT3ZX, 0BBT4ZX	
	Excision and resection	32.28, 32.29, 32.3x	0B5K8ZZ, 0B5L8ZZ, 0B5M8ZZ, 0BBK8ZZ, 0BBL8ZZ, 0BBM4ZZ, 0BBM8ZZ, 0B5K0ZZ, 0B5K3ZZ, 0B5K7ZZ, 0B5L0ZZ, 0B5L3ZZ, 0B5L7ZZ, 0B5M0ZZ, 0B5M3ZZ, 0B5M7ZZ, 0BBK0ZZ, 0BBK3ZZ, 0BBK7ZZ, 0BBL0ZZ, 0BBL3ZZ, 0BBL7ZZ, 0BBM0ZZ, 0BBM3ZZ,	31640, 31641, 32440-32525, 32657, 32663

© 2023 Koroukian SM et al. *JAMA Network Open.*

			0BBM7ZZ, 0BBC4ZZ, 0BBD4ZZ, 0BBF4ZZ, 0BBG4ZZ, 0BBH4ZZ, 0BBJ4ZZ, 0BBK4ZZ, 0BBL4ZZ, 0BTH4ZZ	
Breast	Biopsy	85.11, 85.12	0H9T3ZX, 0H9T7ZX, 0H9T8ZX, 0H9U3ZX, 0H9U7ZX, 0H9U8ZX, 0H9V3ZX, 0H9V7ZX, 0H9V8ZX, 0H9W3ZX, 0H9W7ZX, 0H9W8ZX, 0H9WXZX, 0H9X3ZX, 0H9X7ZX, 0H9X8ZX, 0H9XXZX, 0HBT3ZX, 0HBT7ZX, 0HBT8ZX, 0HBU3ZX, 0HBU7ZX, 0HBU8ZX, 0HBV3ZX, 0HBV7ZX, 0HBV8ZX, 0HBW3ZX, 0HBW7ZX, 0HBW8ZX, 0HBWXZX, 0HBX3ZX, 0HBX7ZX, 0HBX8ZX, 0HBXXZX, 0HBY3ZX, 0HBY7ZX, 0HBY8ZX, 0H9T0ZX, 0H9U0ZX, 0H9V0ZX, 0H9W0ZX, 0H9X0ZX, 0HBT0ZX, 0HBU0ZX, 0HBV0ZX, 0HBW0ZX, 0HBX0ZX, 0HBY0ZX	19100, 19101
	Excision and resection	85.20–85.23, 85.4x	0H5T0ZZ, 0H5T3ZZ, 0H5T7ZZ, 0H5T8ZZ, 0H5U0ZZ, 0H5U3ZZ, 0H5U7ZZ, 0H5U8ZZ, 0H5V0ZZ, 0H5V3ZZ, 0H5V7ZZ, 0H5V8ZZ, 0HBT0ZZ, 0HBT3ZZ, 0HBT7ZZ, 0HBT8ZZ, 0HBU0ZZ, 0HBU3ZZ, 0HBU7ZZ, 0HBU8ZZ, 0HBV0ZZ, 0HBV3ZZ, 0HBV7ZZ, 0HBV8ZZ, 0HBT3ZZ, 0HTT0ZZ, 0HTU0ZZ, 0HTV0ZZ, 07T50ZZ, 0HTT0ZZ, 07T60ZZ, 0KTH0ZZ, 0KTJ0ZZ, 07T70ZZ, 07T80ZZ, 07T70ZZ, 07T90ZZ	19125, 19126, 19160, 19162, 19180-19240
Prostate	Biopsy	60.11, 60.12	0V903ZX, 0V904ZX, 0V907ZX, 0V908ZX, 0VB03ZX, 0VB04ZX, 0VB07ZX, 0VB08ZX, 0V900ZX, 0VB00ZX	55700, 55705, 76360
	Excision and resection	60.2x, 60.3, 60.4, 60.5, 62.4x	0V507ZZ, 0V508ZZ, 0VB07ZZ, 0VB08ZZ, 0VT07ZZ, 0VT08ZZ, 0VT00ZZ, 0VT00ZZ, 0VT04ZZ, 0VT30ZZ, 0VT34ZZ, 0VTC0ZZ, 0VTC4ZZ, 0VT90ZZ, 0VT94ZZ, 0VTB0ZZ, 0VTB4ZZ	52601, 52612–52650, 55801, 55831, 54520, 54530, 55810–55815, 55840–55845
Colorectal	Biopsy	45.25, 45.26, 48.24, 48.25	0D9E3ZX, 0D9E4ZX, 0D9E7ZX, 0D9E8ZX, 0D9H3ZX, 0D9H4ZX, 0D9H7ZX, 0D9H8ZX, 0D9N3ZX, 0D9N4ZX, 0D9N7ZX, 0D9N8ZX, 0DBE3ZX, 0DBE4ZX, 0DBE7ZX, 0DBE8ZX, 0DBH3ZX, 0DBH4ZX, 0DBH7ZX, 0DBH8ZX, 0DBN3ZX, 0DBN4ZX, 0DBN7ZX, 0DBN8ZX, 0DDE3ZX, 0DDE4ZX, 0DDE8ZX, 0DDH3ZX, 0DDH4ZX, 0DDH8ZX, 0DDN3ZX, 0DDN4ZX, 0DDN8ZX, 0D9E0ZX, 0D9H0ZX, 0D9N0ZX, 0DBE0ZX, 0DBH0ZX, 0DBN0ZX, 0D9P3ZX, 0D9P4ZX, 0D9P7ZX, 0D9P8ZX, 0DBP3ZX, 0DBP4ZX, 0DBP7ZX, 0DBP8ZX, 0DDP3ZX, 0DDP4ZX, 0DDP8ZX, 0D9P0ZX, 0DBP0ZX	44389, 45305, 45331, 45390, 76360

© 2023 Koroukian SM et al. *JAMA Network Open.*

	Excision and resection	45.4x, 45.7x, 45.8x, 48.3x, 48.41, 48.49, 48.5x, 48.6x	0DBE0ZZ, 0DBE3ZZ, 0DBE7ZZ, 0DBE4ZZ, 0DBE8ZZ , 0D5E4ZZ, 0D5E8ZZ, 0D5E0ZZ , 0D5E3ZZ, 0D5E7ZZ, 0DTH0ZZ, 0DTH7ZZ, 0DTH8ZZ, 0DTF0ZZ, 0DTF7ZZ, 0DTF8ZZ, 0DTK0ZZ, 0DTL0ZZ, 0DTL7ZZ , 0DTL8ZZ, 0DTLFZZ, 0DTG0ZZ , 0DTG7ZZ, 0DTG8ZZ , 0DTGFZZ, 0DTN0ZZ, 0DTN7ZZ, 0DTN8ZZ, 0DTNFZZ, 0DBE8ZZ, 0DBGFZZ, 0DBLFZZ, 0DBMFZZ, 0DBNFZZ , 0DTMFZZ, 0DTE4ZZ, 0DTE0ZZ, 0DTE7ZZ , 0DTE8ZZ , 0D5P0ZZ, 0D5P3ZZ, 0D5P4ZZ, 0D5P7ZZ, 0D5P8ZZ, 0DBP3ZZ , 0DBP7ZZ, 0DBP8ZZ, 0DBP4ZZ , 0DBP8ZZ , 0DTP7ZZ, 0DTP8ZZ, 0DTP0ZZ, 0DTP4ZZ, 0D1N0Z4, 0DTP0ZZ , 0DTN4ZZ , 0D1N4Z4 , 0DTP4ZZ , 0DBP0ZZ, 0DTP7ZZ , 0DTP8ZZ	44110, 44392–44394, 45160–45180, 45308, 45309, 45315, 45320, 45333, 45338, 45339, 45383–45385, 44140–44160, 45110–45121
	Bypass surgery	46.01, 46.03, 46.1x, 46.2x	0D190Z4, 0D194Z4, 0D198Z4, 0D1A0Z4, 0D1A4Z4, 0D1A8Z4, 0D1B8Z4, 0D1K0Z4, 0D1K4Z4, 0D1K8Z4, 0D1L0Z4, 0D1L4Z4, 0D1L8Z4, 0D1N0Z4, 0D1N4Z4, 0D1H0Z4, 0D1H4Z4, 0D1H8Z4, 0D1K4Z4, 0D1K8Z4, 0D1L8Z4, 0D1N4Z4, 0D1N8Z4, 0D1N8Z4, 0H87XZZ, 0D1B0Z4, 0D1B4Z4, 0H87XZZ	44310, 44320
Pancreatic	Biopsy	52.11, 52.12, 52.14	0F9G3ZX, 0F9G4ZX, 0F9G8ZX, 0FBG3ZX, 0FBG4ZX, 0FBG8ZX, 0F9G0ZX, 0FBG0ZX, 0F9D3ZX, 0F9D4ZX, 0F9D7ZX, 0F9D8ZX, 0FBD3ZX, 0FBD4ZX, 0FBD7ZX, 0FBD8ZX	43261, 48100, 48102, 76360
	Excision and resection	52.2x, 52.5x, 52.6, 52.7	0F5D8ZZ, 0FBD8ZZ, 0F5D0ZZ, 0F5D3ZZ, 0F5D7ZZ, 0F5G0ZZ, 0F5G3ZZ, 0FBD0ZZ, 0FBD3ZZ, 0FBD7ZZ, 0FBG0ZZ, 0FBG3ZZ, 0FTD0ZZ, 0FTD7ZZ, 0FBG0ZZ, 0FBG3ZZ, 0FBG4ZZ, 0FBG8ZZ, 0DT90ZZ, 0DT94ZZ, 0DT97ZZ, 0DT98ZZ, 0FTG0ZZ, 0FTG4Z, 0DB90ZZ, 0DB93ZZ, 0DB94ZZ, 0DB97ZZ, 0DB98ZZ, 0FBG4ZZ, 0DT90ZZ, 0FTG0ZZ	48120, 48145, 48148, 48140–48144, 48146, 48147, 48149–48155

© 2023 Koroukian SM et al. *JAMA Network Open.*

	Bypass surgery	44.39, 51.36, 51.39, 51.42	0D16079, 0D1607A, 0D160J9, 0D160JA, 0D160K9, 0D160KA, 0D160Z9, 0D160ZA, 0D16879, 0D1687A, 0D168J9, 0D168JA, 0D168K9 , 0D168KA, 0D168Z9, 0D168ZA, 0F190D3, 0F190Z3, 0F194D3, 0F194Z3, 0F150D5, 0F150D6, 0F150D7, 0F150D8 , 0F150D9, 0F150Z5, 0F150Z6, 0F150Z7, 0F150Z8, 0F150Z9, 0F154D5, 0F154D6, 0F154D7, 0F154D8, 0F154D9, 0F154Z5, 0F154Z6, 0F154Z7, 0F154Z8, 0F154Z9, 0F160D5 , 0F160D6, 0F160D7, 0F160D8, 0F160D9 , 0F160Z5, 0F160Z6 , 0F160Z7, 0F160Z8, 0F160Z9 , 0F164D5, 0F164D6, 0F164D7, 0F164D8, 0F164D9 , 0F164Z5, 0F164Z6, 0F164Z7 , 0F164Z8, 0F164Z9, 0F170D5, 0F170D6, 0F170D7, 0F170D8, 0F170D9, 0F170Z5, 0F170Z6, 0F170Z7, 0F170Z8, 0F170Z9, 0F174D5, 0F174D6, 0F174D7 , 0F174D8, 0F174D9, 0F174Z5, 0F174Z6, 0F174Z7, 0F174Z8, 0F174Z9, 0F180D4 , 0F180D5, 0F180D6 , 0F180D7, 0F180D8, 0F180D9, 0F180Z4, 0F180Z5, 0F180Z6, 0F180Z7, 0F180Z8, 0F180Z9, 0F184D4, 0F184D5, 0F184D6, 0F184D7, 0F184D8, 0F184D9, 0F184Z4, 0F184Z5, 0F184Z6, 0F184Z7, 0F184Z8, 0F184Z9, 0F190D4, 0F190D5, 0F190D6, 0F190D7, 0F190D8, 0F190D9 , 0F190Z4, 0F190Z5, 0F190Z6, 0F190Z7, 0F190Z8 , 0F190Z9, 0F194D4, 0F194D5 , 0F194D6 , 0F194D7 , 0F194D8, 0F194D9, 0F194Z4, 0F194Z5, 0F194Z6, 0F194Z7, 0F194Z8, 0F194Z9, 0FC90ZZ	43820, 43825, 47720–47780
All Cancers	Chemotherapy	99.25	3E03305, 3E04305	964XX, 965XX, J7150, J85XX, J86XX, J87XX, J8999, J9XXX, Q0083, Q0084, Q0085
	Radiation	92.2x	D0x, D7x, D8x, D9x, DBx, DDx, DFx, DGx, DHx, DPx, DTx, DUx, DVx, DWx	77401–77499, 77750–77799, G0256, G0261

© 2023 Koroukian SM et al. *JAMA Network Open.*

eTable 3. Multivariable Logistic Regression Analysis for Receipt of Aggressive EOL With Adjusted Odds Ratios (OR) and 95% Confidence Intervals

	Any Aggressive EOL Care	Any Cancer-Directed Treatment	>1 Emergency Department Visit	>1 Hospital Admission	Any ICU Admission	Hospice Entry <3 Days of Life	Death in Hospital
**Nursing Home Status**							
Community-Dwelling	ref	ref	ref	ref	ref	ref	ref
Nursing Home	1.04 (1.02-1.07)	0.57 (0.55-0.58)	0.97 (0.95-1.00)	1.06 (1.02-1.10)	0.82 (0.79-0.84)	0.89 (0.86-0.92)	1.61 (1.57-1.65)
**Age Group**							
65-74	ref	ref	ref	ref	ref	ref	ref
75-84	0.80 (0.70-0.82)	0.77 (0.75-0.79)	0.90 (0.87-0.93)	0.82 (0.79-0.85)	0.84 (0.82-0.87	0.95 (0.91-0.98)	0.90 (0.88-0.92)
≥ 85	0.68 (0.66-0.70)	0.50 (0.49-0.52)	0.84 (0.81-0.88)	0.64 (0.61-0.67)	0.66 (0.64-0.69)	0.90 (0.860.94)	0.82 (0.79-0.85)
**Sex**							
Female	ref	ref	ref	ref	ref	Ref	ref
Male	1.25 (1.22-1.28)	1.19 (1.16-1.23)	1.10 (1.06-1.13)	1.13 (1.09-1.17)	1.21 (1.17-1.24)	1.12 (1.08-1.16)	1.18 (1.15-1.21)
**Race/Ethnicity**							
Non-Hispanic White	ref	ref	ref	ref	ref	ref	ref
Non-Hispanic Black	1.1 (1.06-1.15)	0.79 (0.75-0.83)	1.18 (1.13-1.23)	1.18 (1.11-1.24)	1.26 (1.21-1.32)	0.87 (0.82-0.92)	1.18 (1.13-1.23)
Non-Hispanic API	1.08 (1.03-1.14)	1.03 (0.97-1.10)	1.05 (0.98-1.11)	1.12 (1.03-1.21)	1.4 (1.32-1.48)	0.70 (0.64-0.76)	1.33 (1.26-1.41)
Non-Hispanic AI/AN	1.28 (1.07-1.53	1.03 (0.85-1.25)	1.17 (0.95-1.43)	1.24 (0.96-1.59)	1.02 (0.82-1.25)	1.24 (0.98-1.55)	1.16 (0.96-1.40)
Hispanic	1.05 (1.00-1.11)	1.01 (0.96-1.07)	1.14 (1.08-1.20)	1.07 (1.00-1.15)	1.13 (1.07-1.19)	0.89 (0.82-0.95)	1.08 (1.03-1.14)
**Dual Eligible Status**							
No	ref	ref	ref	ref	ref	ref	ref
Yes	0.92 (0.90-0.95)	0.78 (0.75-0.81)	1.02 (0.98-1.05)	0.84 (0.81-0.88)	0.90 (0.87-0.93)	0.75 (0.72-0.78)	1.10 (1.07 1.13
**Cancer Type**							
Lung	ref	ref	ref	ref	ref	ref	ref
Breast	0.95 (0.91-0.99)	0.62 (0.59-0.64)	0.98 (0.94-1.03)	0.91 (0.85-0.96)	0.97 (0.93-1.02)	1.01 (0.96-1.06)	1.06 (1.02-1.11)
Colorectal	0.76 (0.74-0.79)	0.6 (0.58-0.62)	0.84 (0.80-0.87)	0.82 (0.78-0.86)	0.86 (0.83-0.90)	0.86 (0.82-0.89)	0.95 (0.92-0.98)
Pancreatic	0.72 (0.7-0.75)	0.49 (0.47-0.51)	1.00 (0.96-1.04)	1.00 (0.95-1.05)	0.67 (0.64-0.70)	0.93 (0.88-0.97)	0.73 (0.70-0.76)
Prostate	0.79 (0.76-0.81)	0.55 (0.53-0.57)	0.89 (0.85-0.92)	0.85 (0.81-0.89)	0.84 (0.81-0.88)	0.85 (0.81-0.89)	0.91 (0.88-0.94)
**Year of Death**							

© 2023 Koroukian SM et al. *JAMA Network Open.*

2013	ref	ref	ref	ref	ref	ref	ref
2014	1.00 (0.97-1.04)	0.96 (0.93-1.00)	1.07 (1.03-1.11)	0.99 (0.94-1.04)	1.02 (0.98-1.07)	1.03 (0.98-1.08)	1.04 (1.00-1.07)
2015	1.00 (0.96-1.03)	0.93 (0.89-0.96)	1.08 (1.03-1.12)	0.99 (0.94-1.04)	1.03 (0.99-1.07)	1.01 (0.97-1.06)	1.02 (0.98-1.06)
2016	0.95 (0.92-0.98)	0.84 (0.81-0.87)	1.09 (1.05-1.14)	0.96 (0.91-1.00)	1.00 (0.96-1.04)	1.06 (1.01-1.11)	0.98 (0.94-1.01)
2017	0.96 (0.93-1.00)	0.86 (0.83-0.90)	1.15 (1.11-1.20)	0.98 (0.93-1.03)	1.06 (1.02-1.11)	1.06 (1.01-1.11)	0.95 (0.91-0.98)
**Elixhauser Comorbidity Count**							
≤ 4	ref	ref	ref	ref	ref	ref	ref
≥ 5	2.42 (2.37-2.47)	1.44 (1.4-1.48)	2.27 (2.21-2.34)	3.43 (3.30-3.57)	2.51 (2.44-2.58)	1.54 (1.49-1.59)	2.04 (1.99-2.09)

© 2023 Koroukian SM et al. *JAMA Network Open.*

eFigure 1. Cohort Selection Flowchart With Exclusion/Inclusion Criteria

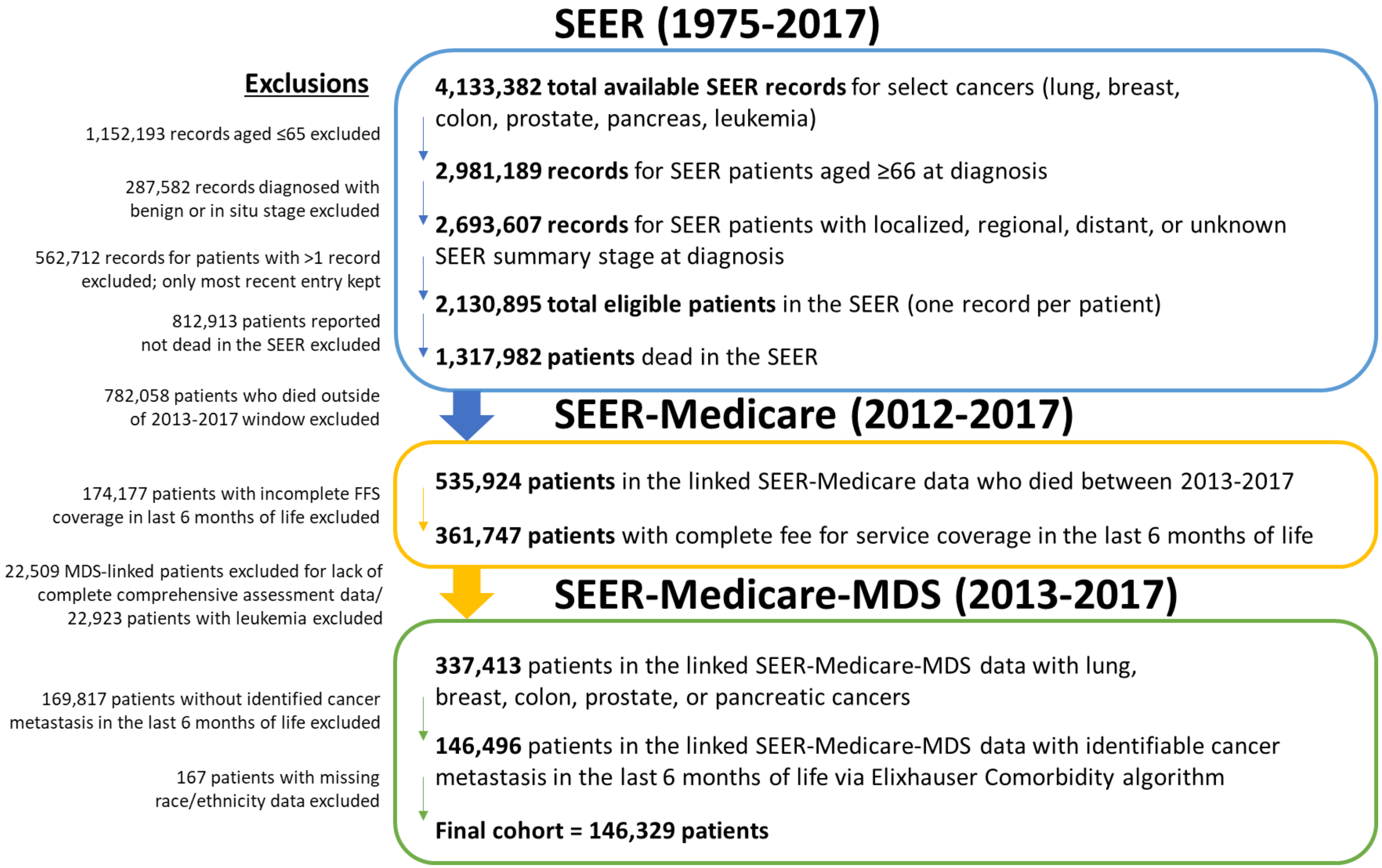

© 2023 Koroukian SM et al. *JAMA Network Open.*

eFigure 2. Sensitivity Analysis of Markers for Receipt of Aggressive EOL Care After Restricting NH Cohort to Only Those With Confirmed NH Enrollment in Last Month of Life

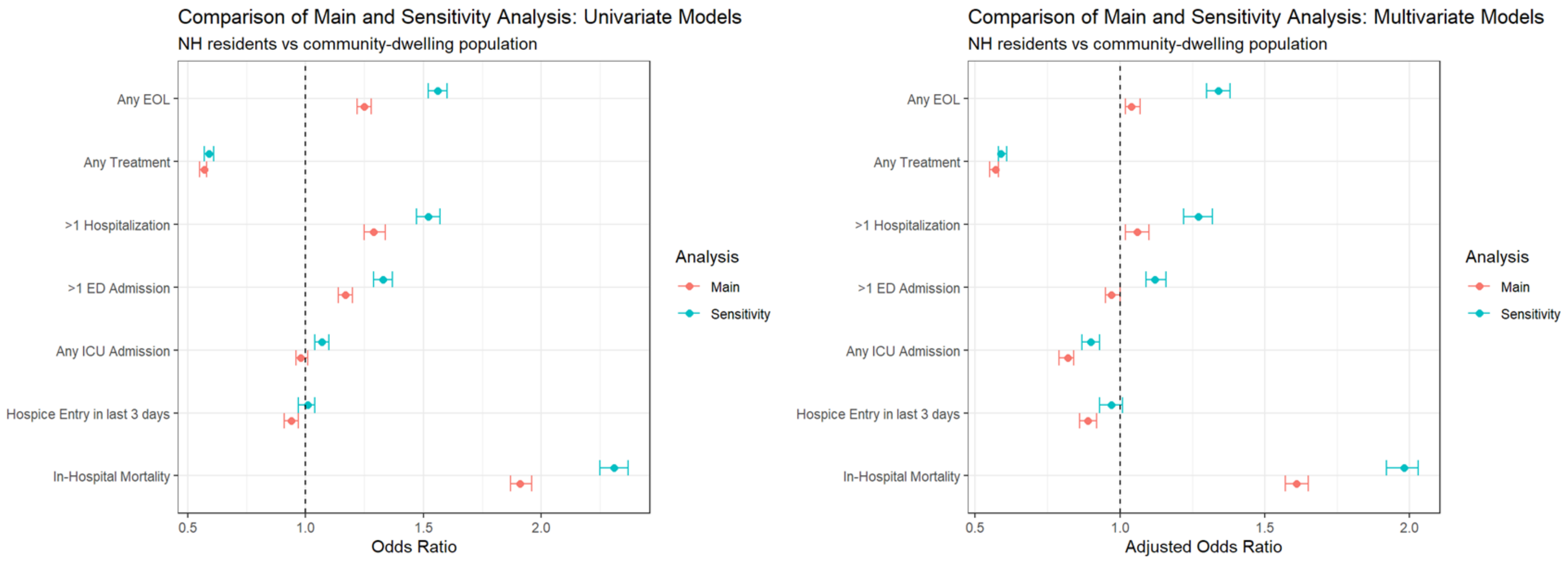

© 2023 Koroukian SM et al. *JAMA Network Open.*
