# Supplementary material for: Incidence of Aggressive End-of-Life Care Among Older Adults With Metastatic Cancer Living in Nursing Homes and Community Settings
Source: JAMA Netw Open. 2023 Feb 22;6(2):e230394. doi: 10.1001/jamanetworkopen.2023.0394 (PMC9947721; doi:10.1001/jamanetworkopen.2023.0394)
Supplement: Supplement 2. — Data Sharing Statement [file jamanetwopen-e230394-s002.pdf]

Data Sharing Statement

Koroukian. Incidence of Aggressive End-of-Life Care Among Older Adults With Metastatic Cancer Living in Nursing Homes and Community Settings. *JAMA Netw Open*. Published February 22, 2023. doi:10.1001/jamanetworkopen.2023.0394

Data
Data available: No

Additional Information
Explanation for why data not available: Data were acquired from Information Management Services, Inc., which develops and maintains the linked Surveillance, Epidemiology and End Results (SEER) and Medicare data linked with the Minimum Data Set (MDS). These data may be made available upon request and are subject to a data users agreement with IMS, Inc. Instructions on how to request SEER-Medicare data linked with MDS can be found at: https://healthcaredelivery.cancer.gov/seermedicare/obtain/requests.html (accessed September 14, 2022)
